# Laparoscopic cholecystectomy *versus* conservative management for uncomplicated symptomatic gallstones: economic evaluation based on the C-GALL trial

**DOI:** 10.1093/bjs/znae293

**Published:** 2025-01-20

**Authors:** Rodolfo A Hernández, Irfan Ahmed, Karen Edwards, Jemma Hudson, Katie Gillies, Rebecca Bruce, Victoria Bell, Alison Avenell, Jane Blazeby, Miriam Brazzelli, Seonaidh Cotton, Bernard Croal, Graeme MacLennan, Peter Murchie, Craig R Ramsay

**Affiliations:** Health Economics Research Unit, University of Aberdeen, Aberdeen, UK; Department of Surgery, NHS Grampian, Aberdeen, UK; Centre for Healthcare Randomised Trials, University of Aberdeen, Aberdeen, UK; Aberdeen Centre for Evaluation, University of Aberdeen, Aberdeen, UK; Aberdeen Centre for Evaluation, University of Aberdeen, Aberdeen, UK; Centre for Healthcare Randomised Trials, University of Aberdeen, Aberdeen, UK; Centre for Healthcare Randomised Trials, University of Aberdeen, Aberdeen, UK; Aberdeen Centre for Evaluation, University of Aberdeen, Aberdeen, UK; Centre for Surgical Research, NIHR Bristol and Western Biomedical Research Centre, University of Bristol, Bristol, UK; Aberdeen Centre for Evaluation, University of Aberdeen, Aberdeen, UK; Centre for Healthcare Randomised Trials, University of Aberdeen, Aberdeen, UK; Clinical Biochemistry, NHS Grampian, Aberdeen, UK; Centre for Healthcare Randomised Trials, University of Aberdeen, Aberdeen, UK; Aberdeen Centre for Evaluation, University of Aberdeen, Aberdeen, UK; Academic Primary Care, Institute of Applied Health Sciences, University of Aberdeen, Aberdeen, UK; Aberdeen Centre for Evaluation, University of Aberdeen, Aberdeen, UK

## Introduction

Worldwide cholecystectomy is the treatment of choice when gallstones are symptomatic^1^. In 2023, about 78 000 cholecystectomies were performed in the UK, costing the National Health Service (NHS) over £200 million^2^. A large UK prospective study found that 10.8% of people experienced complications 30 days after surgery^3^ and up to 40% of people continue to experience pain and abdominal symptoms after surgery^4^. Whilst cholecystectomy was found to be cost-effective in a modelling study^5^, half of people treated conservatively were symptom-free in the long term.

The UK C-GALL trial assessed the clinical and cost-effectiveness of conservative management (CM; pain medication and generic healthy lifestyle advice) compared with laparoscopic cholecystectomy (LC) for preventing recurrent symptoms and complications in adults with uncomplicated symptomatic gallstones^6^. Here, the economic evaluation findings are summarized, with full details reported elsewhere^6^.

## Methods

The C-GALL trial was a parallel-group, multicentre patient randomized superiority pragmatic trial, with 24-month follow-up. Between August 2016 and November 2019, 434 participants were randomized (217 to CM and 217 to LC) from 20 UK centres. The primary clinical outcome was quality of life (QoL) measured by area under the curve (AUC) over 18 months using the Short Form-36 items (SF-36) bodily pain domain^6^.

**Fig. 1:**
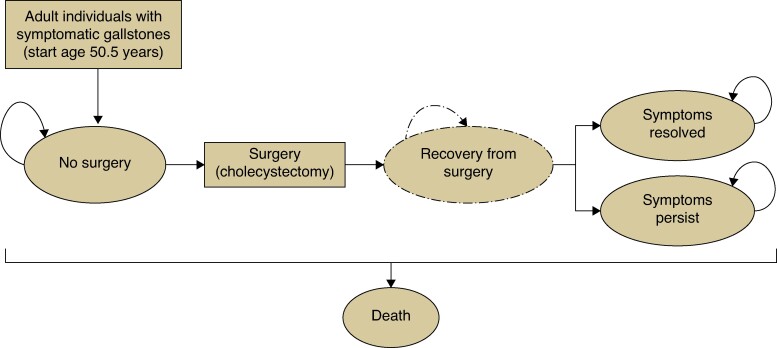
Simplified schematic for the C-GALL Markov model A cohort of individuals with confirmed symptomatic gallstones enter the model in the ‘No surgery’ health state and are assigned to either laparoscopic cholecystectomy or conservative management. Individuals undergoing surgery accrue the cost of the surgical episode and move to the ‘Recovery from surgery’ tunnel state, with an associated reduction in quality of life. On exit from this Markov state, individuals either have their symptoms resolved or not. The absorbing state ‘Death’ can be entered from any other state based on age-specific mortality rates. Individuals receive treatment, as observed in the C-GALL trial, on an intention-to-treat basis, with waiting times being those that occurred in the trial follow-up for the first 24 months. At the start, the mean age was 50.5 years and 71% were women. Monthly Markov cycles and a 10-year time horizon were adopted^6^.

The within-trial (24-month follow-up) and Markov-model (*Fig. 1*) extrapolation economic analyses followed established economic evaluation methods, with prespecified health economics analysis^6^. The UK NHS healthcare system perspective was adopted, with costs expressed in British Pounds Sterling for the 2019–2020 price year (average exchange rate £1 = €1.19, October 2024). Data regarding hospital and primary care resources used were collected and valued using routine cost data^7^. Quality-adjusted life-years (QALYs) were estimated using participants’ responses to the SF-36 questionnaire and Short Form-6 Dimensions (SF-6D) population-norm utilities, with a decrease in QoL associated with need for surgery^8^. Beyond year one, the annual discount rate was 3.5%^9^. Deterministic and probabilistic analyses were conducted to characterize uncertainty.

## Results

At 24 months, 64 (29.5%) participants had cholecystectomies in the CM group and 153 (70.5%) participants had cholecystectomies in the LC group. The mean(s.d.) time in theatre was slightly longer for the CM group (83(49) *versus* 72(42) min) and the mean(s.d.) duration of hospital stay was slightly longer for the CM group (1.4(3.4) *versus* 0.63(1.3) days). Further hospital admissions were experienced by 28 (12.9%) participants in the CM group and 21 (9.7%) participants in the LC group. Based on responses from 96 participants in the CM group and 95 participants in the LC group, 45 and 21 Accident and Emergency admissions were counted, respectively.

Adjusted mean costs and QALYs were higher for the LC group (*Table 1*), producing an incremental cost-effectiveness ratio (ICER) of £55 235 and £78 063 for the 24-month follow-up and modelling extrapolation respectively, above the usual threshold used for UK decision-making (£20 000)^9^.

**Table 1:** Cost–utility analysis results

Intervention	Total cost (£)	Incremental cost (£) (95% credible interval)*	Total QALYs	Incremental QALYs (95% credible interval)*	ICER	Probability of being cost-effective		
						£13 000	£20 000	£30 000
**Within-trial base-case analysis (full cohort; HRG-based costing; multiple imputation)**								
Laparoscopic cholecystectomy (current practice)	2510	–	1.413	–	–	0.011	0.065	0.229
Conservative management	1477	−1033 (−1413,−632)	1.395	−0.019 (−0.056,0.020)	55 235	0.989	0.935	0.771
**Decision-model base-case analysis; 10-year time horizon**								
Laparoscopic cholecystectomy	3020	–	5.907	–	–	0.000	0.016	0.109
Conservative management	2016	−1003	5.894	−0.013	78 063	1.000	0.984	0.891
**Selected sensitivity analysis: decision-model results using C-GALL-adjusted utilities up to 48 months; 10-year time horizon**								
Laparoscopic cholecystectomy	3020	–	5.914	–	–	0.371	0.827	0.975
Conservative management	2016	−1003	5.846	−0.068	14 698	0.629	0.173	0.025

Average exchange rate £1 = €1.19, October 2024. *The incremental cost and QALY for conservative management compared with laparoscopic cholecystectomy up to 24 months post-randomization were estimated using generalized linear regression models, adjusted for minimization factors (centre, age, and sex) and baseline utility score, with multiple imputation implemented for the primary analysis; the ICER was then calculated, with uncertainty surrounding the joint incremental costs and effects characterized using non-parametric bootstrapping^6^. QALYs, quality-adjusted life-years; ICER, incremental cost-effectiveness ratio (incremental cost/incremental QALY); HRG, healthcare resource groups.

## Discussion

CM was less costly than LC, with no significant difference in QALYs. The ICER was high, meaning important potential NHS savings could be achieved, with limited QALY loss, by following CM in the short term. Longer-term modelling suggested that CM might be cost-effective, but there was greater uncertainty due to limited information on subsequent surgeries in both groups, and differences in QoL beyond 24 months could reverse this finding. Sensitivity analysis incorporating longer-term QoL scores reduced potential savings to just £14 698 per QALY lost. The current decision uncertainty could be reduced by long-term follow-up.

In the present study, the modelling extrapolation analyses were underpinned by randomized trial data that were collected prospectively. The pragmatic nature of the C-GALL trial and the intention-to-treat principles facilitate the generalizability of the findings to the patient population routinely treated by the NHS in the UK. However, COVID-19-related NHS-imposed restrictions on elective procedures, such as cholecystectomy, might have favoured the use of CM in both trial groups. A second important limitation relates to the short follow-up and the natural history of gallstone disease. Cholecystectomy might be indicated in the future for participants under CM, as well as those still waiting for surgery in the cholecystectomy group. Schmidt *et al*.^10^ reported on 14-year follow-up for an RCT that randomized 137 participants to observation or cholecystectomy, stating a median time to cholecystectomy for those participants in the observation group of 28 months. In the present study, decision modelling was used to address this limitation, extrapolating the analysis to a 10-year time horizon. Furthermore, there was no difference in the utility score attached to the ‘Symptoms resolved’ and ‘Symptoms persist’ Markov health states after surgery. As the proportion of cholecystectomies was higher in the cholecystectomy group, reduced QoL due to symptoms after surgery would make cholecystectomy less cost-effective and, therefore, the results of the present study are conservative.

To the authors’ knowledge, this is the first economic evaluation alongside an RCT comparing cholecystectomy with CM. An economic analysis by Latenstein *et al*.^11^ was conducted alongside the SECURE RCT, which compared usual care with a restrictive surgical strategy. Participants in the restrictive strategy arm were offered cholecystectomy when they fulfilled all five prespecified criteria: they experienced severe pain attacks; the attacks lasted 15–30 min or longer; the pain was located in the epigastrium or right upper quadrant; the pain radiated to the back; and they had a positive pain response to simple analgesics. Latenstein *et al*.^11^ found that the restrictive strategy was cost saving, but resulted in fewer pain-free patients at 12 months. Latenstein *et al*.^11^ found a relatively small difference in QALYs, consistent with the results of the present study; however, Latenstein *et al*.^11^ did not conduct an incremental cost–utility analysis, as they understood that the near-zero absolute difference in QALYs would easily render the incremental analysis into positive or negative infinity.

In summary, the cost–utility analysis based on the C-GALL trial participant data suggests that CM is cost-effective at 24-month follow-up. However, the extrapolation over 10 years introduced uncertainty over this short-term result and could in some scenarios reverse the findings. The current decision uncertainty could be reduced with long-term follow-up of the C-GALL trial participants. Further research should focus on identifying the cohort of patients that should be routinely offered surgery.

## Data Availability

The data generated from this study are not publicly available, but may be made available, upon specific request to the corresponding author.
